# Guidance Cue Netrin-1 and the Regulation of Inflammation in Acute and Chronic Kidney Disease

**DOI:** 10.1155/2014/525891

**Published:** 2014-06-03

**Authors:** Punithavathi Ranganathan, Riyaz Mohamed, Calpurnia Jayakumar, Ganesan Ramesh

**Affiliations:** ^1^Vascular Biology Center, Georgia Regents University, Augusta, GA 30912, USA; ^2^Department of Medicine/Vascular Biology Center, CB-3702, Georgia Regents University, 1459 Laney-Walker Boulevard, Augusta, GA 30912, USA

## Abstract

Acute kidney injury (AKI) is a common problem in the hospital setting and intensive care unit. Despite improved understanding, there are no effective therapies available to treat AKI. A large body of evidence strongly suggests that ischemia reperfusion injury is an inflammatory disease mediated by both adaptive and innate immune systems. Cell migration also plays an important role in embryonic development and inflammation, and this process is highly regulated to ensure tissue homeostasis. One such paradigm exists in the developing nervous system, where neuronal migration is mediated by a balance between chemoattractive and chemorepulsive signals. The ability of the guidance molecule netrin-1 to repulse or abolish attraction of neuronal cells expressing the UNC5B receptor makes it an attractive candidate for the regulation of inflammatory cell migration. Recent identification of netrin-1 as regulators of immune cell migration has led to a large number of studies looking into how netrin-1 controls inflammation and inflammatory cell migration. This review will focus on recent advances in understanding netrin-1 mediated regulation of inflammation during acute and chronic kidney disease and whether netrin-1 and its receptor activation can be used to treat acute and chronic kidney disease.

## 1. Introduction

Acute kidney injury (AKI) has now replaced the old terminology acute renal failure. Clinically AKI is defined as a rapid decline in kidney function resulting in failure to maintain fluid, electrolyte, and acid-base homoeostasis. The incidence of AKI is increasing which is further complicated by lack of effective therapies to reduce or prevent it from happening. AKI has a frequency of 1–9% in hospital inpatients and over 40% in critically ill patients in the intensive care units if sepsis is present [1–3]. Similarly, chronic kidney disease due to diabetes contributes to a significant amount of mortality and morbidity. In the United States, approximately 20 million people or 7% of the population are estimated to have diabetes and the incidence of diabetes is growing. Diabetes has become the primary cause of end-stage renal disease (ESRD). Approximately 44% of new patients entering dialysis in the USA are diabetics [4, 5]. Studies in animals and human suggested that acute and chronic kidney diseases are inflammatory disease and inflammatory mediators play a major role in tissue injury seen in both forms of kidney disease [6–14].

Inflammation is defined as a cellular response to injurious stimulus which is classified into two broad categories: (1) nonsterile inflammation and (2) sterile inflammation. Nonsterile inflammation usually occurs during infection whereas sterile inflammation usually occurs without infection but during tissue injury due to surgery, metal toxicity, ischemia, drugs, or chemicals. Similar to nonsterile inflammation in response to infection, sterile inflammation also exhibits a similar manifestation such as vasodilation, edema, leukocyte infiltration into the tissues, and cellular damage by apoptosis and necrosis [15, 16]. However, the initial events that elicit and control the response can be very different between sterile and nonsterile inflammation. The dying and dead cell often release intracellular contents that are not usually exposed to immune systems such as ATP, uric acid, heat shock proteins, high mobility group of proteins, nucleic acid, and many others [17–21] which may act as ligands for pattern recognition receptors on the cell surface of innate immune system and adjacent cells causing activation of those cells. Activated innate immune cells and adaptive immune cells release cell damaging reactive oxygen and nitrogen species, proteases, and cytokines [15, 22]. Although these damaging molecules are beneficial during infection to clear pathogen and during tissue regeneration process or wound healing, uncontrolled release of these molecules during early stages of tissue injury often causes excessive damage to normal tissue which can lead to further reduction in organ function [7, 12–14, 23].

Cells have defensive protective mechanism often activated in parallel with the inflammatory response to counteract the damaging effects of innate immune cells. These cytoprotective molecules include anti-inflammatory cytokines (IL-10 and TGF*β*1), neuronal guidance cues netrins, adenosine, heme oxygenase, and others. Inadequate response or downregulation of these counteracting pathways may exacerbate inflammatory response and tissue injury. But at the same time, complete suppression of the inflammatory response is detrimental to system survival as it may experience susceptibility to infection or reduced capacity to regenerate injured tissues.

## 2. Inflammation in Acute Kidney Injury

**Figure 1:**
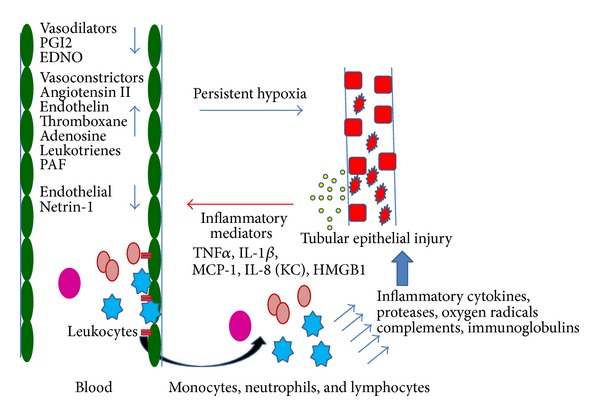
Inflammation in acute kidney injury. Ischemia reperfusion or nephrotoxin causes tubular epithelial stress or necrosis which releases inflammatory mediators. These inflammatory mediators activate endothelial cells and expression of adhesion molecules (II) such as ICAM-1 induces leukocyte migration into the interstitium and release of vasoconstrictor. At the same time inflammation downregulates anti-inflammatory molecules such as netrin-1 and endothelial nitric oxide. Infiltrated white blood cells such as neutrophils, monocyte, and T cells release cytokine, chemokines, proteases, and oxygen radicals cause further damage to the epithelium. All these events lead to vasoconstriction and persistent hypoxia which contributes to further tissue damage.

Animal and human studies suggest that AKI such as ischemic kidney disease and cisplatin induced kidney damage is inflammatory disease mediated by both innate and adaptive immune systems [12–14, 24, 25]. The role of immune cells in ischemia reperfusion injury of the kidney has been known for more than a decade. Using gene knockout, specific cell depletion, adoptive transfer of immune cells, and generation of bone marrow chimera model, the role of specific immune cell type in ischemia reperfusion injury was determined. These studies indicated the involvement of both the adaptive and innate immune systems in AKI [7, 12, 26–31]. Most components of innate (macrophage, neutrophils, and NKT) [26, 30, 32] and adoptive (CD4^+^ T cells and B cells) [31, 33] immune systems have been shown to mediate ischemia reperfusion injury of the kidney. Depletion of macrophage [30, 34], neutrophils [35], CD4 T cells [31], B cells [33], or NKT cells (with NK1.1 mAb) [26] is protective against ischemia reperfusion injury of the kidney, whereas depletion of regulatory T cells (Treg) exacerbates AKI [36]. Recent studies suggest that the administration of Treg or adenosine treated dendritic cells before ischemia is also protective [36, 37]. In contrast to the above observations, opposing views on the role of adaptive immune systems in ischemia reperfusion injury have also been documented [38, 39]. For example, depletion of neutrophils in T and B cell deficient mice is not protective against ischemia reperfusion injury of the kidney [39]. Similarly, dendritic depletion is protective against ischemia reperfusion injury of the kidney [25], whereas dendritic cell depletion exacerbates cisplatin induced AKI [40]. Many of T cell, macrophage, and neutrophil secreted cytokines (IL-6, TNF*α*, IL-17, and IFN*γ*) [14, 26, 41, 42] and chemokines (MCP-1, KC, and MIP-2) [9, 43, 44] are also shown to mediate renal ischemia reperfusion injury and cisplatin induced AKI. In addition, toll-like receptors 2 and 4 are shown to be critical mediators of AKI [10, 29]. The conclusion drawn from these studies is that every cell type of immune system or their secreted components (cytokines and chemokines) play a critical role in mediating ischemia reperfusion injury of the kidney (Figure 1). The challenge is to translate these findings into useful therapies in human. Depletion of all cell types of immune systems for therapeutic purpose will be difficult and also may leave the patients with immune deficiency. Depletion of one specific cell type may not be protective or may offer only marginal protection. Moreover, in most of these studies in animal models of AKI, immune cell depletion was performed prior to reperfusion injury. However, in the hospital setting this is not possible as AKI development is difficult to predict but can be diagnosed early once it develops. Therefore, effectiveness of depletion after reperfusion injury is initiated has never been studied. Moreover, in the model system used for ischemia reperfusion injury such as bilateral renal pedicle clamping, injury happens so rapidly and depletion of immune cells after initiation of injury could be problematic. Recent animal study suggests that TLR4 ligand HMGB1, cytokines such as IL-1*α* and -*β*, IFN*γ*, KC, GM-CSF, MIP-1*α*, and VEGF peak as early as 1 minute after reperfusion [45]. In addition, recent studies also suggest that AKI is a systemic inflammatory disease that affects other organs as well. Therefore, therapy based on molecules which regulate inflammation by suppressing immune cell activation and migration into injured organs will be effective in treating ischemic kidney disease and other forms of AKI. Moreover, these molecules will suppress inflammation and leave immune system intact; thereby possible immunodeficiency will be avoided. However, knowledge on such molecules which can negatively regulate innate and adaptive immune systems is lacking. Understanding such molecule, like netrin-1, will provide new direction to treat inflammatory ischemic kidney disease. This review will focus on one such counteractive anti-inflammatory pathway netrin-1 and its receptor UNC5B in the regulation of inflammation during acute (ischemic, drug induced) and chronic (diabetes) kidney injury.

## 3. Netrins and Their Receptors

The name netrin-1 is derived from Sanskrit Netr, meaning “one who guides.” Studies in the nematode* Caenorhabditis elegans* identified genes required for circumferential axon guidance [46, 47]. One of the genes identified,* unc-*6, encoded a secreted protein with sequence homology to laminins [48]. In 1994, using commissural axon outgrowth from explants of embryonic rat dorsal spinal cord as a functional assay, two proteins were purified from homogenates of embryonic chick brain and discovered to be homologous to UNC-6 [49]. They were named netrin-1 and netrin-2. Netrin-1 is a laminin-related molecule initially discovered as a diffusible molecule produced by a ventral structure in the developing spinal cord, the floor plate, which attracts commissural axons [49]. Netrin-1 was thus shown to act as a chemoattractive or chemorepulsive cue for many migrating axons and neurons during the development of the nervous system [50, 51].

Five netrins have been identified in vertebrates so far: netrin-1 has been identified in chicken [52], mouse [53],* Xenopus* [54], zebrafish [55, 56], and humans [57]; netrin-2 in chickens [52]; and netrin-3 in humans (NTN2L) [58] and mouse [59]; netrin-4 in mouse, human, rat,* Xenopus*, and chicken [60]. In addition, two glycosylphosphatidyl inositol- (GPI-) modified, membrane anchored netrin family members (netrin G1 and netrin G2) were discovered. The membrane-anchored netrin Gs are reported to have evolved independently of the secreted netrins [61, 62] and are found only in vertebrates. All netrins are composed of approximately 600 amino acids and have a molecular mass of approximately 70 kilodaltons. Netrins 1, 2, 3, and 4 are all structurally related to the short arms of laminin *γ* chains and contain a laminin VI domain and three EGF like repeats similar to the laminin V domain (V-1, V-2, and V-3); they also contain a positively charged heparin-binding COOH-terminal domain termed domain C [49, 63].

**Figure 2:**
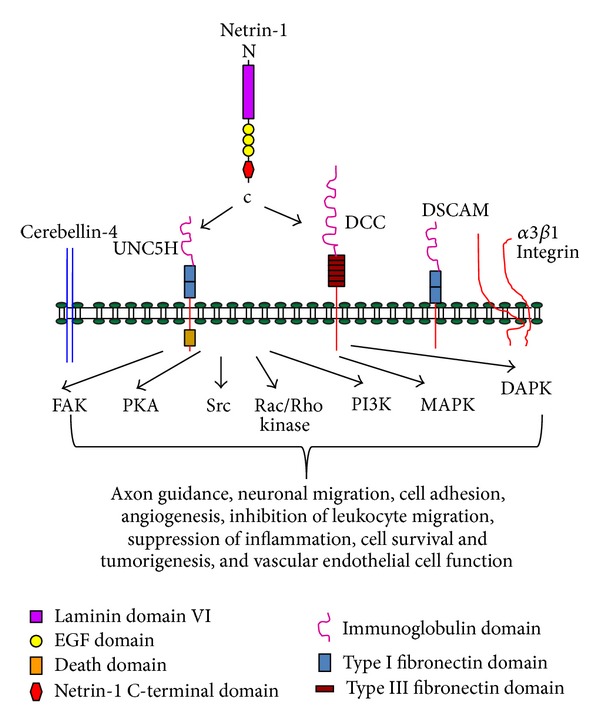
Netrin-1 receptor signaling and known functions. Netrin-1 is known to bind several receptors which include uncoordinated 5 H (UNC5H1-4), deleted in colorectal cancer (DCC), down syndrome cell adhesion molecule (DSCAM), integrins (*α*3*β*1 & *α*6*β*3), and cerebellin-4. Binding of netrin-1 to these receptors is known to activate several signaling pathways including focal adhesion kinase (FAK), protein kinase A (PKA), src kinase, Rac/Rho kinase, phosphor inositol 3-phosphate activated kinase (PI3K), and mitogen activated kinase (MAPK). Death associated protein kinase (DAPK) activation occurs in the absence of ligand binding. Activation of these pathways was associated with changes in cellular function listed in the figure.

Netrin-1 effect is regulated by the interaction with its main receptors, deleted in colorectal cancer (DCC) [53, 63] and uncoordinated family member 5 (UNC5A-D or UNC 5H 1–4) [64, 65] (Figure 2). In addition, recently, four additional receptors have been identified which include Down syndrome cell adhesion molecule (DSCAM) [66], integrin *α*3*β*1 and *α*6*β*3 [67, 68], cerebellin 4 (CBLN4) [69] (Figure 2), and a controversial receptor adenosine receptor (A2bR) [70]. Structurally, netrin-1 resembles the extracellular matrix protein laminin and is thought to have derived from the laminin *γ* chain. Netrin-1 comprises a globular domain (VI) at the amino terminus which is followed by three epidermal growth factor (EGF) repeats, namely, V1, V2, and V3. Domains VI and V bind to the fibronectin type III domains of DCC and immunoglobulin domains of UNC-5 families of netrin-1 receptors [65], which are followed by the positively charged C terminal domain (C). Domain V is the most highly conserved across the netrins. Given the homology between DCC and neogenin, it is likely that netrin-1 also binds the fibronectin type III domains of neogenin. However, recent studies suggest that neogenin has specific ligand called repulsive guidance molecule (RGM) [71]. Netrin-4 is also known to bind neogenin [72]. Netrin-1 C-terminal domain contains the integrin recognition sites including the RGD motif recognized by *α*3*β*1. The C domain also contains binding sites for membrane glycolipids and extracellular matrix components including heparan sulfate proteoglycans [73].

Netrin-1 signaling is complex and is not always attractive or stimulatory. Binding of netrin-1 to DCC induces axonal attraction [70], whereas binding to the UNC5 receptor family causes repulsion [64, 74]. Binding of netrin-1 to DCC and UNC5B induces activation of multiple pathways which include MAPKs, PKC, src, PI3 kinase, Rac and Rho kinase, focal adhesion kinase, and many others (Figure 2) [60, 65, 75, 76].

Netrin-1 receptors DCC and UNC5B are also called dependence receptors. They transmit signal even in the absence of ligand. When the ligand is available, these receptors transduce a positive signaling pathway leading to cellular proliferation, differentiation, migration, or survival. However, in the absence of their ligand, these receptors are not inactive, like “classical” receptors, but rather induce a “negative signaling” that triggers caspase dependent apoptotic cell death [77, 78]. DCC has classical death domain but not UNC5B. However, both receptors are capable of inducing apoptosis in the absence of ligand netrin-1. Dependence receptor concepts and activity are described in detail elsewhere [76, 77, 79, 80]. In contrast to the secreted netrins, netrin-Gs bind transmembrane proteins called the netrin-G ligands (NGL) and netrin-Gs do not appear to interact with DCC, neogenin, or the UNC5 proteins [61, 81].

Mouse kidney expresses netrin-1, netrin-3, and netrin-4 and their receptors UNC5B, UNC5C, and neogenin [82, 83]. The expression of another receptor mRNA is minimal or negligible. Immunolocalization studies had determined that DCC expression is not detectable in kidney epithelium (unpublished, Ramesh G), whereas UNC5B is expressed only in proximal tubular epithelial cells and vascular endothelial cells. Neogenin is expressed in all segments of nephron but in the basolateral surface similar to netrin-4 localization [82, 83]. UNC5C is localized in the distal tubular epithelium [82]. Similarly, the expression of netrin-1 is undetectable in immune cells, whereas UNC5B and to some extent UNC5A expression but not other receptor expressions were detected in immune cells [13]. The expression of integrins and DSCAM is not determined in the kidney.

## 4. Regulation of Inflammation by Netrin-1 in Acute Kidney Injury

Netrin-1 was identified as neuronal guidance cue, directing neurons and their axons to targets during development of the nervous system [84]. While netrin-1 is primarily thought of as an axon guidance cue, guidance is unlikely to be its only function since expression studies have shown that netrins are widely expressed outside the nervous system including vascular endothelial cells [83, 85]. Vascular endothelial cells play critical barrier for leukocyte activation and migration into organs by producing repellent factors to leukocytes such as netrin-1. Migration of inflammatory cells to the site of injury is a critical cellular response to initiate the removal of dead cells and induce a regeneration response. However, inappropriate excessive activation and migration of these cells into organs can also result in tissue destruction, and this abnormal influx is thought to be the mechanism involved in many ischemic injuries of organs [13, 31, 86–89]. Barriers to aberrant immune cell activation and migration may exist, for example, the production of immunosuppressive, chemorepellent molecules from the endothelium, and epithelial cells. Downregulation of these chemorepellent factors such as netrin-1 during organ injury may exacerbate inflammation [83, 85, 88]. Initial evidence to support this idea came from in vitro studies. Ly et al. in 2005 [85] demonstrated that netrin-1 inhibits leukocyte migration in response to chemotactic signal. In the same study, it was also shown that sepsis downregulated netrin-1 in endothelial cells which may contribute to increased transendothelial migration of leukocyte. Receptor that mediates in vivo anti-inflammatory function of netrin-1 and whether netrin-1 is also effective in other disease models and tissues was unknown at that time.

Netrin-1 is expressed in many tissues including brain, lung, heart, liver, intestine, and kidney. Kidney is among the highest netrin-1-expressing organs studied [13, 83, 85]. Despite its high expression, the role of netrins in kidney physiology and pathophysiology was unknown. First clue came from animal model of ischemia reperfusion injury of the kidney. We proposed that endothelial netrin-1 represents yet another homeostatic protein whose dysregulation after ischemia contributes to the development of organ failure. Studies from our lab demonstrated some novel insights regarding the roles of netrins in IR injury of the kidney. I/R injury was associated with dramatic changes in abundance and localization of netrin-1. Within 3 h after reperfusion, netrin-1 protein expression was highly induced in tubular epithelial cells with a decrease in peritubular vasculature. By 24 h of IR, netrin-1 expression was seen only in tubular epithelial cells. However, netrin-1 and netrin-4 mRNA were downregulated despite increased protein expression in epithelial cells at early hours after reperfusion. Moreover, the distribution of netrin-1 changed from being primarily endothelial to being primarily epithelial [83]. Although netrin-1 returned to normal by 48 h after reperfusion, netrin-4 mRNA was persistently downregulated. Similarly, ischemia reperfusion downregulated circulating netrin-1 levels. Moreover, endothelial cells that are subjected to hypoxia also downregulated netrin-1 expression [83]. The functional significance of these changes was assessed by administering recombinant netrins intravenously to mice before I/R injury. Netrin-1 exerted a dose-dependent protection against renal dysfunction [83]. Interestingly, netrin-1 administration reduced neutrophil infiltration into the kidney and the expression of cytokines and chemokines in the kidney. Netrin-4 infusion had no effect on renal function or injury. Since exogenous netrin-1 administration was protective, we speculated that the observed loss of netrin-1 in endothelial cells, rather than increase in epithelial netrin-1, contributes to ischemia reperfusion injury. Since netrin-1 mRNA did not increase in concert with protein abundance, netrin-1 production by epithelial cells may be regulated at the translational level. Subsequently, another study reported a similar anti-inflammatory effect of netrin-1 using whole body hypoxia model [88] and ischemia reperfusion injury of the kidney using a partial netrin-1 knockout (heterozygous knockout) [90]. Interestingly, proximal tubular epithelial cell specific overexpression also protected kidney through suppressing epithelial cells apoptosis [91]. Subsequent studies from our laboratory and other laboratories had demonstrated that administration of netrin-1 reduced inflammation and tissue injury in many different disease models such as acute colitis [92, 93], acute lung injury [94], peritonitis [95], and cisplatin induced AKI [96].

## 5. Regulation of Inflammation by Netrin-1 in Chronic Kidney Disease

**Figure 3:**
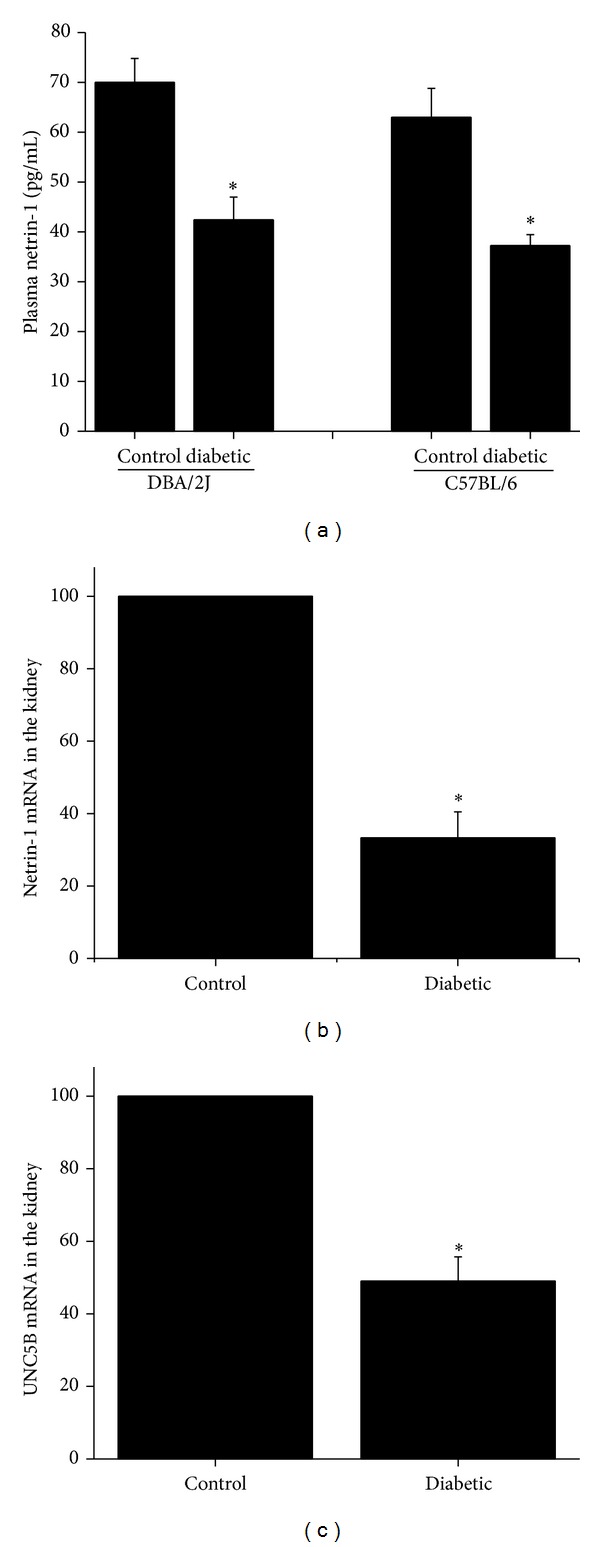
Diabetes downregulates endogenous anti-inflammatory protective pathways. (a) Diabetes (8 weeks after STZ administration) downregulated plasma netrin-1. *P < 0.001 versus nondiabetic control. *n* = 5. Netrin-1 in plasma was quantified by ELISA. (b) and (c) RT-PCR analysis of netrin-1 and UNC5B expression in control and 12-week diabetic mouse. Diabetes downregulated netrin-1 (C) and UNC5B (G) expression in the kidney. *P < 0.001 versus control. *n* = 5.

Diabetic nephropathy is a complex chronic disease involving interactions between kidney cells (i.e., endothelial cells, podocytes, and epithelial cells) and immune cells. Its course is accelerated by failure of endogenous renal protective mechanisms in the chronic diabetic milieu. Between 30 and 50% of prevalent patients who are on hemodialysis (HD) have elevated serum levels of inflammatory markers such as C-reactive protein and IL-6 [97, 98]. In some patients, this elevation is chronic, and in some, it is intermittent and generally is associated with breakthrough processes. Furthermore, on many occasions, HD sessions trigger inflammation in a way that is not always identifiable with the conventional markers. Inflammatory markers are powerful predictors of mortality after adjustment for other risk factors [97, 99]. Inflammation also is responsible for other mortality risk factors, such as anemia, malnutrition, vascular disease, and left ventricular hypertrophy [97, 100]. The guidance cue netrin-1 and its receptor UNC5B represent an endogenous anti-inflammatory pathway that is widely expressed in kidney cells and immune cells. The regulation of netrin-1 and its receptor in the kidney during diabetes is not clearly defined. Moreover, its role in the regulation during dialysis is unknown. Previous studies from our lab determined that netrin-1 protein is induced in epithelial cells and excreted in urine during diabetes both in animal model and in humans [101, 102]. In vitro studies had shown that hyperglycemia downregulates netrin-1 expression whereas addition of high concentration of protein BSA induced netrin-1 in the same cells [103]. This induction appears to be a translational mechanism. In addition, diabetes downregulated circulating levels of netrin-1 in two different genetic models. Consistent with downregulation of plasma netrin-1, both netrin-1 and UNC5B mRNA were found to be significantly downregulated in diabetic kidney as compared to control (Figure 3). No change was seen for UNC5B, while netrin-1 protein was undetectable in macrophage RAW264.7 cells (not shown) with/without 30 mM glucose. These findings demonstrate that diabetes downregulates the endogenous netrin-1-UNC5B anti-inflammatory pathway systemically and in the kidney, while proteinuria may upregulate netrin-1 in renal epithelial cells in response to injury [103]. Moreover, overexpression of netrin-1 in tubular epithelial cells before the start of the disease process suppressed diabetes induced increase in infiltration of neutrophils and macrophages, chemokine expression, albuminuria, and tubular epithelial cell apoptosis in kidneys [104]. In addition, diabetes induced a large increase in the excretion of prostaglandin E2 (PGE2) in urine, which was suppressed in netrin-1 transgenic mice. Netrin-1-induced suppression of PGE2 production was mediated through suppression of NF*κ*B-mediated cyclooxygenase-2 (COX-2) in renal tubular epithelial cells [104]. Using a different approach, Eunyong Tak et al. also showed the elevated levels of albuminuria, glomerular filtration rate, and severe loss of kidney function in partial netrin-1 deficient (Ntrn1 ± mice) diabetic mice, whereas administration of recombinant netrin-1 was associated with attenuated albuminuria and improved histological score for diabetic nephropathy compared to control mice [105]. These results suggest that netrin-1 is a major regulator of inflammation and apoptosis in diabetic nephropathy and may be a useful therapeutic molecule for treating chronic kidney diseases such as diabetic nephropathy.

## 6. UNC5B Receptor Mediates Netrin-1 Anti-Inflammatory Effects

Infiltration of neutrophils and monocytes is one of the hallmarks of tissue injury in ischemia reperfusion injury of the kidney. Neutrophils were shown to mediate renal ischemia reperfusion injury [26, 106, 107]. Cytokines, such as IL-17 and IFN-*γ*, produced by neutrophils are known to mediate renal ischemia reperfusion injury [26, 106]. Since only UNC5B receptor expression was seen in a significant amount in leukocytes, we [13] investigated the effect of UNC5B neutralization on netrin-1 mediated suppression of inflammation and chemokine and cytokine production in renal IR injury. The response of the innate immune system to tissue injury is very rapid as seen by a rapid influx of monocytes and neutrophils into the kidney subsequent to renal IRI. Administration of recombinant netrin-1 before or after renal ischemia-reperfusion reduced kidney injury, apoptosis, monocyte and neutrophil infiltration, and cytokine (IL-6, IL-1*β*, and, TNF*α*) and chemokine (MCP-1, macrophage-derived cytokine, monokine-induced IFN-*γ*, keratinocyte-derived chemokine, and chemokine with 6 cysteines) production [13]. Analysis of different netrin-1 receptors expression on leukocytes showed very high expression of UNC5B but not UNC5C, UNC5D, neogenin, or deleted in colorectal cancer [13]. Expression of UNC5A was low. Neutralization of UNC5B receptor reduced netrin-1-mediated protection against renal ischemia-reperfusion injury and increased monocyte and neutrophil infiltration as well as serum and renal cytokine and chemokine production with increased kidney injury and renal tubular cell apoptosis. T cells and dendritic cells content in the kidney were not significantly changed with/without netrin-1 in response to ischemia reperfusion. However, still they may mediate reperfusion injury as shown in many excellent studies [25, 31, 108]. In contrast to the innate immune system, the activation of adaptive immune cells typically takes longer. However, Ag-independent activation of the adaptive immune system is possible [109] which will take only few minutes. For example, a hypoxic condition augments cytokine production in CD4 T cells [110]. CD4 T cell cytokines such as IL-6, IL-17, TNF-*α*, and IFN-*γ* contribute to the pathogenesis of IRI of kidney, liver, lung, and intestine [106, 111–114]. The CD4 T cell cytokines are also known to regulate the expression of other soluble mediators of inflammation and function of leukocytes, including monocytes and neutrophils. Investigation into netrin-1's effect on CD4 T cell stimulation showed suppression of Th1/Th2/Th17 cytokine (IL-2, IL-6, IL-10, IL-13, IL-17, IFN-g, IL-4, and TNF*α*) production in vitro which was inhibited with UNC5B receptor neutralization [13]. These results had demonstrated that netrin-1 acting through UNC5B receptor reduces renal ischemia-reperfusion injury and its associated renal inflammation. This observation was also confirmed using tissue specific UNC5B knockout mice. Deletion of a single allele or proximal tubular epithelial cell specific deletion of both alleles of UNC5B exacerbated AKI due to ischemia reperfusion and cisplatin. Moreover, neutrophil infiltration and inflammatory cytokine production dramatically increased in the kidney suggesting that UNC5B signaling regulates injury and inflammatory response in AKI [115].

It was not clear whether netrin-1 mediated suppression of neutrophil and monocyte infiltration into the kidney and inhibition of cytokine production are due to its direct effect on neutrophils and monocytes or acting through T cells. This was clarified using a T and B cell deficient mice (RAG-1 knockout). Administration of recombinant netrin-1 to both WT and RAG-1 knockout mice protected kidney from IR injury in both WT and RAG-1 knockout mice by suppressing inflammation, infiltration of neutrophils, and apoptosis [116], suggesting that the effect of netrin-1 on neutrophil infiltration is independent of T and B cells.

## 7. Netrin-1 Regulates Inflammation through NF*κ*B and COX-2/PGE2 Pathways

**Figure 4:**
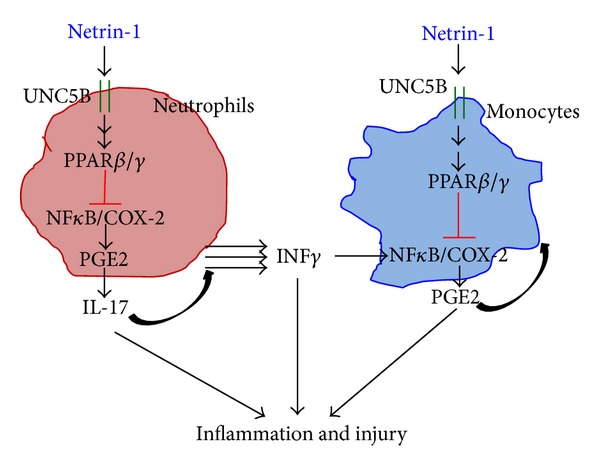
Netrin-1 regulates neutrophil and monocyte mediated inflammation through suppression of COX-2 expression and PGE2 production. Netrin-1 acting through UNC5B receptor induces the activation of PPAR*γ*. Activation of PPAR*γ* and other signaling pathways like PI3K causes suppression of I*κ*B degradation and inactivation of NF*κ*B transcription factor. NF*κ*B activation is required for COX-2 induction. In the absence of NF*κ*B activation COX-2 expression and PGE2 production was suppressed. This leads to reduced IL-17 production and IFN*γ* production thereby suppresses inflammation and tissue injury during ischemia reperfusion injury.

The signaling pathway through which netrin-1 suppresses cytokine production is not clear. Netrin-1 is known to activate adenylate cyclase/cAMP pathways [117, 118]. cAMP/protein kinase A-mediated activation of CREB protein suppresses cytokine production from immune cells [119, 120]. Therefore, it is possible that netrin-1-mediated activation of adenylate cyclase/cAMP pathways may inhibit the production of proinflammatory mediators. Recent studies did indicate the increase in cAMP production by netrin-1 in immune cells [85, 94, 95]. However, it is not clear as there are additional pathways that exist for netrin-1 to mediate anti-inflammatory effects. Studies from our lab identified that netrin-1 could regulate inflammation through suppression of NF*κ*B activation through suppression of I*κ*B degradation [104]. Since NF*κ*B is a known regulator of cyclooxygenase pathways, the relation between netrin-1 and COX-2 pathways was examined. Administration of netrin-1 suppressed ischemia reperfusion induced COX-2 expression in tubular epithelial cells as well as neutrophils and macrophages which was associated with reduction in prostaglandin E2 and thromboxane B2 excretion in urine [116]. This suggested that netrin-1 may regulate inflammation through inhibition of COX-2 expression in neutrophils and monocyte. Flow cytometry analysis showed that over 86% of Gr-1 positive neutrophils and 80% of F4/80 positive monocyte/macrophage were positive for COX-2 expression. Very few CD4 T cells were seen in the kidney and 60% of them were positive for COX-2 expression [116]. In addition, neutrophils were colocalized for COX-2, confirming that COX-2-expressing infiltrating cells were indeed neutrophils and macrophages. In vitro studies showed that IFN*γ* and LPS-induced COX-2 and PGE2 production were suppressed in netrin-1 treated macrophages. Moreover, IFN*γ* induced increase in MCP-1 and IP-10 production was inhibited by netrin-1 [116]. The netrin-1 mediated suppressive effect on chemokine production was abolished when PGE2 is added to the culture, suggesting that the netrin-1 effect is at the level of COX-2 expression. IL-17 production in neutrophils in response to ischemia is known to initiate IFN*γ* production and infiltration into kidney [106]. Moreover, IFN*γ* also regulates IL-17 production in neutrophils. Previous studies have shown that PGE2 increases IL-17 production in both Th17 cells and neutrophils [121]. We determined whether IL-17-induced IFN*γ* production is dependent on COX-2-mediated PGE2 and that netrin-1 suppresses this pathway, thereby suppressing IFN*γ* production and neutrophil infiltration into ischemic kidney. When neutrophils were stimulated with IL-17, COX-2 expression and IFN*γ* production were increased which was inhibited by netrin-1. The suppressive effect of netrin-1 on IFN*γ* production was abolished by the addition of PGE2, suggesting that the netrin-1 effect is at the level of PGE2 production but is not at the level of its activity [86, 116]. These results suggested that netrin-1 may regulate COX-2 expression through the inhibition of NF*κ*B activation (Figure 4).

## 8. Netrin-1 Regulates Macrophage Polarization through PPAR Pathways

Macrophages express distinct patterns of surface receptors and metabolic enzymes in response to different stimuli and these ultimately generate the diversity in macrophage functions and phenotypes. Broadly, there are two distinct polarization states of macrophages, M1 and M2, that have been characterized [122–124]. However, more recent studies suggest a spectrum of macrophage polarized state depending on the stimulus characterized by distinct sets of genes that are activated with each stimulus [125]. However, whether such a spectrum of macrophage polarization exists in renal diseases is unknown. LPS and IFN*γ* can promote macrophage differentiation to a “classical” or M1 phenotype [126]. The M1 activation pattern is associated with tissue destruction and inflammation and is responsible for upregulating proinflammatory cytokines and increasing the production of reactive nitrogen species and reactive oxygen species [127]. In contrast, the “alternative” or M2 activation phenotype of macrophages is induced in response to IL-4 and IL-13. M2-polarized macrophages dampen the inflammatory process by producing anti-inflammatory factors, such as IL-10 and TGF-*β*1. M2 macrophages also upregulate mannose receptor (MR), C type 2 (Mrc2c), IL-1 receptor antagonist (IL-1RA), and scavenger receptors, such as cluster of differentiation 36 (CD36), as well as increased expression of arginase-1 (Arg-1). The M2 phenotype is thought to promote tissue repair after inflammation and/or injury [122–124, 127].

Improper macrophage activation is pathogenically linked to various metabolic, inflammatory, and immune disorders. Therefore, regulatory proteins controlling macrophage activation have emerged as important new therapeutic targets. Previous studies from our lab demonstrated that netrin-1 regulates inflammation and infiltration of monocytes and ameliorates ischemia reperfusion-induced kidney injury [13, 83, 116]. In subsequent studies, we determined whether netrin-1 regulates the phenotype of macrophages and the signaling mechanism involved in the macrophage polarization. Overexpression of netrin-1 increased expression of arginase-1, IL-4, and IL-13 and decreased expression of COX-2 in spleen and kidney, indicating a phenotypic switch in macrophage polarization toward an M2-like phenotype [86]. Moreover, flow cytometry analysis showed a significant increase in mannose receptor-positive macrophages in spleen as compared to wild type. In vitro, netrin-1 increased expression of M2 markers (MR, arginase-1, and IL-10) but suppressed expression of M1 marker (iNOS and IL-1*β*) in both peritoneal macrophages and RAW264.7 cells [86]. Moreover, netrin-1 suppressed IFN*γ*-induced M1 polarization and production of inflammatory mediators such as IL-6 and IP-10. Adoptive transfer of netrin-1-treated macrophages suppressed inflammation and kidney injury against ischemia reperfusion. Interestingly, netrin-1 activated three anti-inflammatory pathways in macrophage. These include the PPARs (plasmid has a combination of PPAR*α*, PPAR*β*/*δ*, and PPAR*γ* response elements), glucocorticoid response element (GRE), and retinoic acid response elements (RXRs). PPARs are members of a nuclear-hormone-receptor superfamily; they transduce a wide variety of signals, including environmental, nutritional, and inflammatory events into a defined and ordered set of cellular responses at the level of gene transcription. Various types of fatty acid metabolites of arachidonic acid can bind and activate PPARs. Recent evidence has indicated an important role for PPARs in the control of various types of the inflammatory response [128]. These functions are mediated by several mechanisms which include the abilities of the PPARs to transrepress the activities of many activated transcription factors (nuclear factor-*κ*B (NF-*κ*B)), signal transducers and activators of transcription (STATs), activator protein 1 (AP1) and nuclear factor of activated T cells (NFAT), transcriptional upregulation of NF*κ*B inhibitor kappa B (I*κ*B), and the ability of PPAR-RXR heterodimers to inhibit phosphorylation of the MAPK (JNK and p38) cascade [128, 129]. Apart from the transcriptional activation of PPARs, netrin-1 induced the PPAR*γ* ligand 15-deoxy-Δ^12,14^-prostaglandin J_2_ in the presence of TLR4 activation in macrophage cell line [86]. Neither TLR4 activation alone nor netrin-1 increases 15-deoxy-Δ^12,14^-prostaglandin J_2_ levels in macrophages [86]. Administration of netrin-1 suppressed ischemia reperfusion injury, which was abolished by PPAR*γ* antagonist. In vitro, addition of netrin-1 strongly induced PPAR*β* and -*γ* expression and activation suggesting a role for PPAR*γ* in mediating the netrin-1 effects in vitro and in vivo. This is further supported by in vitro studies where netrin-1 treatment increased the expression of M2 markers (MR and IL-10) whereas IFN*γ* treatment induced M1 markers (COX-2, iNOS, and IL-6). Addition of netrin-1 with IFN*γ* suppressed M1 marker expression but enhanced M2 marker expression as compared to cells treated with IFN*γ* alone [86]. Importantly, when cells were treated with PPAR*β* and PPAR*γ* antagonist along with netrin-1 and IFN*γ*, the M2 polarizing effects of netrin-1 were abolished suggesting the important role of PPAR pathways in mediating netrin-1 effects in macrophages [86].

## 9. Conclusion

**Figure 5:**
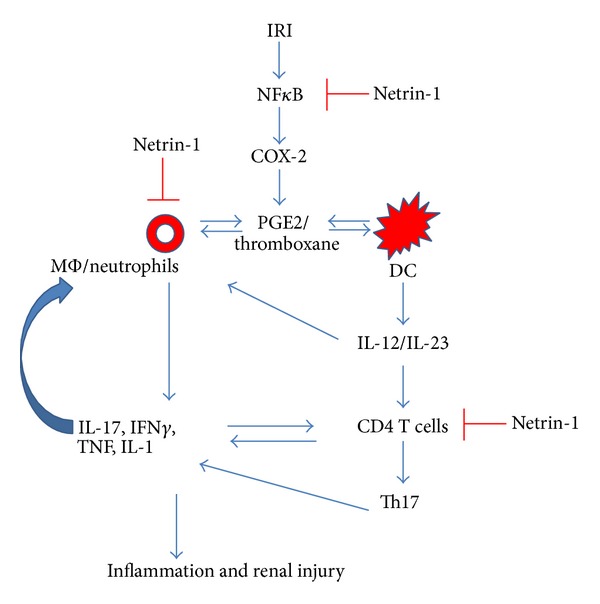
Pathways of inflammatory mediators and their regulation of netrin-1 during ischemia reperfusion injury of the kidney. Ischemic insults initiate inflammation in the kidney through activation of NF*κ*B mediated COX-2 expression and prostaglandin E2/thromboxane production. These mediators, acting through an autocrine or paracrine mechanism, stimulate the production of cytokine and chemokines from monocyte (MΦ), neutrophils, and dendritic cells (DC). T cells may be activated through DC or direct activation by soluble mediators released from other cells or receptor mediated mechanism. Activation and release of soluble mediators from these white blood cells cause vascular leakage, edema, hypoxia, and tubular epithelial cell damage which will be manifested as kidney dysfunction. Netrin-1 reduces the severity of these events by acting at multiple levels through downregulation of COX-2 expression and suppression of inflammatory mediators production.

Guidance cue netrin-1 is a versatile molecule and its role beyond axon guidance is implicated which includes regulation of immune cell migration, cytokine production, macrophage polarization, and regulation of cyclooxygenase-2 pathways. Studies performed in acute and chronic kidney disease as well as disease of other organs have clearly indicated that netrin-1 is a useful therapeutic agent to control inflammation and tissue injury acting at multiple levels (Figure 5). Future studies will identify new pathways that are regulated by netrin-1 in immune and nonimmune cells. Development of netrin-1 based therapies or small molecule that can activate its receptor UNC5B will be able to treat not only kidney disease but also the inflammatory disease of other organs as well.
